# Perioperative Management of Antiplatelet-Drugs in Cardiac Surgery

**DOI:** 10.2174/157340309788166688

**Published:** 2009-05

**Authors:** Raquel Ferrandis, Juan V. Llau, Ana Mugarra

**Affiliations:** Department of Anaesthesiology and Critical Care Medicine, Hospital Clínic Universitari, València, Spain

## Abstract

The management of coronary patients scheduled for a coronary artery bypass grafting (CABG), who are receiving one or more antiplatelet drugs, is plenty of controversies. It has been shown that withdrawal of antiplatelet drugs is associated with an increased risk of a thrombotic event, but surgery under an altered platelet function also means an increased risk of bleeding in the perioperative period. Because of the conflict recommendations, this review article tries to evaluate the outcome of different perioperative antiplatelet protocols in patients with coronary artery disease undergoing CABG.

## 1. INTRODUCTION

The incidence of coronary artery disease (CAD) is high and increasing. First treatment of occlusive coronary disease involves percutaneous revascularization and many times one or more stents placement. Any percutaneous coronary intervention causes trauma to the vessel wall, rendering the endoluminal surface thrombogenic and thus, dual anti-platelet therapy (mostly aspirin and clopidogrel) is currently recommended [1, 2].

When these patients are scheduled for coronary artery bypass grafting (CABG), the traditional recommendation has been to stop antiplatelet drugs between 7 to 10 days prior to surgery [3]. But, withdrawal of aspirin in patients with CAD has been associated with a 2 to 4-fold increase in the risk of death and myocardial infarction [4], being the major independent predictor of stent occlusion [5, 6]. Thus, the anaesthesiologist faces the dilemma of stopping the antiplatelet treatment to avoid bleeding and risking postoperative stent thrombosis, or to maintain the antiplatelet therapy perioperatively to avoid the stent thrombosis, so risking major blood loss and increased transfusion rate.

We lack scientific evidence on the optimum perioperative therapy in such a situation. Because of the conflict recommendations, we undertook this systematic review of the literature to evaluate the outcome of different perioperative antiplatelet protocols in patients with CAD undergoing CABG.

## 2. MAIN CHARACTERISTICS OF ANTIPLATELET DRUGS

The well established current indications of antiplatelet drugs (APD) are shown in Table **1** [7, 8].

All of them are capable to inhibit platelet function, particularly activation and subsequent aggregation, although they make this effect through different ways showing different antiaggregant power (Table **2**) [9, 10]. APD can be classified into four groups:

1. **Adenosin diphosphate (ADP) receptor antagonists**, such as the thienopyridine drugs ticlopidine and clopidogrel, which reach their peak of activity after 3-5 days, producing a prolonged antiaggregant effect (7-10 days) due to its long  half-life. The inhibitory  effects  of clopidogrel could be attained earlier by using 300 or 600 mg loading dose. Moreover, 600 mg double bolus has been shown to achieve greater platelet inhibition than conventional single loading doses [11].
2. **GPIIb/IIIa receptor antagonists**, of exclusively intravenous use, which are more powerful, albeit with a shorter-lasting action (24 h): eptifibatide, abciximab, tirofiban.
3. **Drugs that increase the intraplatelet levels of AMPc**. The best known agent in this group is dipyridamole (moderate antiaggregant effect lasting about 24 hours). Other drugs are the I-2 prostaglandin (epoprostenol) and its analog iloprost, both used by intravenous route with a brief antiaggregant effect (< 3 h).
4. **Inhibitors of ciclooxygenase 1 enzyme (COX-1)**. The best known representatives are acetylsalicylic acid (ASA) and non-esteroidal anti-inflammatory drugs (NSAIDs). ASA is the most deeply studied one and its antiaggregant effect takes place with the irreversible blockade of COX-1, so its action lasts throughout all the life of the platelet (7-10 days). Nevertheless, from the third or fourth day usually there is enough number of platelets to guarantee suitable haemostasis. The NSAIDs also produce inhibition of platelet aggregation by a similar mechanism to the ASA, although there are two important differences: firstly the blocking effect of the COX-1 is reversible, thus once the drug has been eliminated from the circulation, the platelet function is restored; secondly, there is a great difference between the different NSAIDs in their capacity to inhibit COX-1 and, consequently, in its platelet antiaggregant action.

Actually the field of the indications of use of the APD is being continuously updated. In cardiologic patients, some recent questions of development of the APD deserved to be highlighted:

- The role of the aspirin in the primary prevention has extended its prescription based on related factors of cardiovascular and/or neurological risk. Moreover the combination of two APD drugs (mainly ASA and clopidogrel) in high risk patients is a practice more and more extended [9].
- Dual antiplatelet therapy has to be maintained at least 12 months after drug eluting stent placement and elective surgery postponed; if surgery is necessary, at least ASA should be maintained throughout the periperative period and the patient operated under its antiaggregant effect [12]. Probably, in this patient a specific protocol of antiaggregation in type, combination and duration of APD need to be applied [13, 14].
- The interindividual response to the APD is evident and it does not seem that a valid universal pattern of antiaggregation for all the patients exists.

Then, we face different type of patients who benefit from antiplatelet therapy (Table **3**) [15, 16]. From our point of view, we could distinguish two groups: patients with chronic treatment who are scheduled for cardiac surgery and patients who require urgent surgery, most of them with double antiaggregation.

## 3. CARDIOVASCULAR RISK AFTER ANTIPLATELET DRUGS WITHDRAWAL

The antiplatelet therapy in patients at high risk of occlusive vascular events reduce the combined outcome of any serious vascular event by about one quarter, non-fatal myocardial infarction by one third, non-fatal stroke by one quarter, and vascular mortality by one sixth (with no apparent adverse effect on other death) [17]. Aspirin has been the most widely studied APD, with doses of 75-150 mg daily at least as effective as higher daily doses. Keeping patients on aspirin prior to surgery may help to attenuate the inflammatory response during the operative period and may also reduce cardiovascular events while awaiting surgery [18].

As we have previously described, the platelet inhibition achieved with aspirin, although irreversible for target platelets, lasts until a significant pool of new platelets is produced. Nevertheless, a complete recovery of platelet aggregation has been observed by day 3 (in 50% healthy young men) [19] which seems due in part of a biological platelet aggregation “rebound phenomenon”. In fact, after aspirin discontinuation, the recovery of cyclooxigenase activity may occur rapidly, with a heterogeneous synthesis of thromboxane A_2_ by fresh platelets [20], which may have possible hazardous effects in patients with cardiovascular disease [21]. Nevertheless, none of the guidelines support the assessment of platelet reactivity by using point-of-care devices.

Many studies have been recently published describing the cardiovascular risk of perioperative APD withdrawal, most of them in non-cardiac surgery, but we did not find any study comparing the cardiovascular risks of preprocedural APD withdrawal directly against APD continuation. Instead of it, we collect some studies that report the frequency of APD (aspirin) withdrawal preceding acute cardiovascular syndrome (Table **4**) [4, 22-24].

Talking about clopidogrel, most guidelines would recommend cessation for 5-7 days before surgery. The CURE study [25] and its sub-analyses show that cessation of clopidogrel in these patients and for this time period is associated with a 1% increase in the risk of myocardial infarction. In non-surgical patients, with acute coronary artery syndrome, the first 90-day interval after stopping treatment with clopidogrel was associated with a significantly higher risk of adverse events (incidence rate ratio of 1.98) [26].

## 4. BLEEEDING RISK WITH PERIOPERATIVE TREATMENT OF ANTIPLATELET DRUGS

There are many clinical studies comparing periprocedural-bleeding risks with and without aspirin. Excluding studies on cardiac surgery, aspirin multiplied baseline-bleeding rate by a factor of 1.5 [24], although mortalities possibly caused by bleeding occurred only after transurethral prostatectomy [27] and intracranial neurosurgery [28].

Focus on cardiac surgery, the results are heterogeneous but there seems to have an increase of bleeding with a tendency to need more transfusion requirements in patients under the effect of aspirin [29-35]. Thus, in a recent systematic review of randomized and observational studies [36], pre-operative aspirin maintenance was associated with a significant increase in the volume of post-operative bleeding (mean difference 114 ml) and transfusion requirements (mean difference 0.34 units), with not significantly difference in the rates of reoperation; with the subgroup analysis, the authors conclude that this bleeding could be minimized by the use of aspirin doses less than 325 mg/day. Another metanalysis [37] shows similar results, highlighting that the rate of platelet transfusion was similar in both groups.

Bleeding complications and transfusion requirements could be lower if the CABG surgery is performed off-pump, and patients under the effect of aspirin seems to be at less risk for bleeding or for reoperation due to postoperative haemorrhage [38], even if aspirin is associated with clopidogrel and not discontinued within 2 days of surgery (no differences with the discontinuation more than 6 days before surgery or between 2 and 5 days before surgery) [39]. Moreover, some articles have associated preoperative aspirin maintenance with a decreased risk of mortality in CABG patients without significant increase in haemorrhage, blood product requirements, or related morbidities [40], even with aspirin usage within the 5 days preceding surgery [41].

Thus, some surgical and patients characteristics as older age, smaller body mass index, non-elective cases, 5 or more distal anastomosis are more important risk factors for re-exploration for bleeding after CABG than aspirin ingestion, which is consistent with other previous studies [42, 43].

The other common drugs used as antiplatalet agent are thienopyridines that antagonist irreversibly the platelet adenosine diphosphate. It has showed to increase bleeding after CABG in many articles [44-48], although there is another report in which the maintenance of clopidogrel does not increase bleeding or transfusion requirements [49]. The optimal waiting period after last clopidogrel administration is not known but appears to be at least 5 days before CABG [50]; if the patient need to be antiaggregated near before cardiac surgery, probably the best option is to use low-dose aspirin perioperatively, once clopidogrel has been discontinued [51]. Finally, it has been published that a combined preoperative treatment with heparin infusion could prevent the increased blood loss associated to the administration of clopidogrel, which may have been attributable to a conservation of coagulation factors, as evidenced by the increased plasma fibrinogen concentrations with combined prophylactic treatment [52].

Other drugs that have become increasingly common are the intravenous GP IIb/IIIa platelet receptor antagonist (tirofiban, eptifibate and abciximab). While eptifibatide and tirofiban have a competitive binding and are rapidly cleared, with an almost recovered platelet function in about 4 hours [53], abciximab causes prolonged and irreversible effect, with inhibition of platelet function and aggregation lasting 24-48 hours [53]. Transfusion of platelets rapidly reverses the inhibitory effects of abciximab [54], but it is of very little utility during infusion or suddenly after eptifibatide or tirofiban. No data are available in the literature on the impact of tirofiban or eptifibatide treatment on emergency CABG; however their short half-life and short-lasting action after stopping the infusion is a potential advantage for the performance of CABG with reduced risk of bleeding [55-58], so no delay in surgery has been recommended [59]. In patients treated with abciximab, delaying emergency or urgent CABG for 12 hours has been recommend [59, 60], but other authors suggest rapid discontinuation of abciximab infusion and undelayed intervention [61, 62]. In any case, platelet transfusion should be considered only when increased bleeding is encountered (not prophylactically), and only after cessation of cardiopulmonary bypass [63].

### 4.1. Methods to Avoid Bleeding

In cardiac surgery, we should also take care of a variety of methods intended to minimize perioperative transfusion: preoperative autologous donation, intra- and postoperative cell salvage, and the use of drugs, such as aprotinin (a protease inhibitor), desmopressin (that induces the release of factor VIIIvW) and tranexamic acid (TXA) and epsilon-aminocarproic acid (EACA) (that mainly inhibit plasmin binding to fibrin).

Many studies have shown that aprotinin decreased the number of allogenic transfusions after cardiac surgery and the proportion of patients requiring reoperation because of bleeding [64]. Recent publications, however, have questioned the safety of this agent, reporting not only an increase in renal impairment but an increased risk of long-term mortality following CABG surgery [65-67]. The FDA and the EMEA have recently suspended its use, pending of a complete analysis of a randomized prospective trial in Canada (BART trial), which has shown similar results in terms of mortality.

Desmopressin has not shown a statistically significant effect on reducing the proportion of patients receiving transfusion after CABG [64], but its efficacy seemed to vary depending on the use of aspirin. While some authors have shown that desmopressin reduced the postoperative blood loss and the transfusion requirements in patients treated with aspirin within 7 to 5 days before surgery compared to placebo [68-70], no effect was found in patients treated within 2 days before CABG [71].

The TXA decreases the portion of patients receiving allogenic blood, but has not statistically significant effect on reoperations because of bleeding [64], both in on- and in off-pump surgery [72]. This effect is no consistent in all the studies, excluding patients with APD, prophylactic use of TXA did not result in any significant decrease in postoperative bleeding in one study [73], but reduced postoperative bleeding and fibrinolysis in another [74]. In patients treated with aspirin, the administration of a single dose of TXA (30 mg/kg) immediately before cardiopulmonary bypass significantly reduced postoperative bleeding and inhibited fibrinolysis [75].

There are very few studies with EACA that have not shown any statistically significant effect [64].

### 4.2. Monitors of Platelet Function Perioperatively 

The current standard of care for perioperative coagulation monitoring consists of a platelet count and prothrombin (PT) and activated partial thromboplastin (aPTT) times, omitting platelet function. However, these routine tests are insensitive predictors of bleeding and perioperative changes in platelet count shows poor correlation with changes in platelet function [76]. It would be helpful in the perioperative management of APD to have a haemostatic test to identify the patients at bleeding risk because of platelet dysfunction related with the administration of any APD. 

Several tests could be used to assess the platelet function. *Optical platelet aggregometry* is considered at present the reference assay for diagnosis of platelet disorders [12], although it is not completely standardized, the laboratory work up is complex and it is not possible to be performed immediately before the surgery. The *Platelet Function Analyser* (PFA-100) explores the platelet adhesive capacity, measuring the closure time taken for a platelet plug to occlude an aperture in a membrane impregnated with collagen and epinephrine or ADP [77]; ASA and clopidogrel have been shown to prolong this closure time, but without evident correlation with a perioperative bleeding. The *Plateletworks^TM^ analyser* measures the percentage of aggregation of whole blood before and after the exposure to ADP; its results are contradictory when compared with optical aggregometry: good correlation for clopidogrel [78] but of limited use for ASA [79]. *Thromboelastogrphy* (TEG) is a whole blood coagulation monitor, which can demonstrate the alteration of platelet aggregation, but is unable to detect the defects that occur with ASA or demonstrate the ADP blockade caused by clopidogrel.

Unfortunately any of these tests has good correlation with perioperative bleeding and further clinical investigations are necessary in this field, although they can help us to reduce the rate of reoperation for bleeding (TEG), in part by helping to differentiate surgical from nonsurgical bleeding [80], or to improve appropriate platelet transfusion (PFA-100) [81].

## 5. GUIDELINES AND RECOMMENDATIONS

The management of patients under the effect of antiaggregant agents scheduled for cardiac surgery is a major topic of interest and concern for all perioperative caregivers. Many recommendations could be found in the available published papers [8, 13, 14, 36, 37, 51, 82-85] and they could be summarized as follows:

### 1. Patient Treated with Aspirin

- Aspirin should be maintained in patients at high risk for arterial thrombotic complications. The optimal dose of aspirin ranges between 75 and 325 mg and in the perioperative period, in the majority of patients, it would be enough the maintenance of low-dose of aspirin.
- In the case of high risk of bleeding, some drugs that decrease postoperative bleeding, as TXA or EACA (with limited evidence to support the use of one agent over the other) could be used; desmopresine might be considered preoperatively only in patients with acquired or inherited defects in primary haemostasis detected by abnormal point-of-care test, as PFA-100.

### 2. Patient Treated with Clopidogrel

- If the patient is on treatment with clopidogrel and needs to be antiaggregated near before cardiac surgery, probably the best option is to discontinue clopidogrel (at least 5 days before surgery) and use low-dose aspirin perioperatively (75-125 mg daily).
- Dual antiplatelet therapy is associated with too high bleeding risk. If it is mandatory to maintain this protocol before surgery (probably only in patients with a drug-eluting stent implanted less than 12 months ago), and because of the concerns about premature discontinuation of clopidogrel in these very high thrombotic risk patients, several algorhythms have been proposed, including the administration of an intravenous glycoprotein IIb/IIIa inhibitor or unfractionated heparin as “bridging therapy”. At present, there is no enough evidence-based date to support this strategy.

### 3. Patient Treated with GP IIb/IIIa Inhibitor

- In emergency surgery, if the patient is under the effect of a glycoprotein IIb/IIIa inhibitor, it might be considered the platelet transfusion (mainly if it is abciximab) if there is too much bleeding; due to the short-acting time of eptifibatide or tirofiban, the delay of surgery is not recommended in the case of previous administration of them.

### 4. Postoperative Treatment

- If aspirin therapy has been interrupted before surgery, it should be administered early after surgery, always within 48 hours after CABG, and preferably within 6 hours after surgery. Dose ranges between 150-325 mg/day; optimal benefit could be reach with 325 mg/day, at least the first year.
- There is no specific recommendation for resuming clopidogrel after surgery, and it seems to have no superiority over aspirin; if it is indicated instead of aspirin, the timing for its administration could be the same as for aspirin, but it is not recommended a loading dose (300-600 mg) if first administration is close after surgery.

### 5. Others

- Blood salvage techniques use must be encouraged, as the devices that conserve blood (intraoperative blood salvage) or the use of autologous blood predonation or normovolaemic haemodilution.
- Platelet transfusion is not indicated as prophylaxis to avoid bleeding, even if the patient is under the effect of aspirin or clopidogrel, and its administration must be reserved if necessary to control excessive bleeding.
- It is necessary an optimal preparation of the patient, avoiding anaemia stimulating the administration of drugs that increase preoperative blood volume, as erythro-poietin in combination with iron.
- Several guidelines recommend a multimodality approach to blood conservation with the setting-up of consensus algorhythms and point-of-care testing.

## 6. CONCLUSIONS

The handicap of management of antiplatelet agents in the perioperative period of cardiac surgery requires close collaboration between cardiologists, surgeons and anaesthesiologists. It is necessary to avoid thrombotic complications maintaining the antiaggregation, but balancing bleeding complications.

Patients treated with long-term APD could be at risk for increased bleeding if it is maintained until surgery, mainly if there are any other outstanding variable as indicator of risk: advanced age, preoperative anaemia, reoperative o complex procedures, emergency operations or non-cardiac patient comorbidities. If the patient is under the effect of one or more of these drugs the associated bleeding risk might be carefully balanced and an alternative antiaggregation protocol could be considered. Moreover, the drugs to minimize bleeding could play an important role and might be in consideration.

The decision to stop the APD some days before surgery faces up to the decision to maintain the treatment up to surgery. The choice for one or the other option should be based on the individual balance between the risk to develop any cardiovascular event if the APD has been withdrawn and the complications associated as result of the major bleeding if the APD has been maintained until the day of the surgery.

Summarising both possibilities and the comments stated above, we know that clopidogrel maintenance prior to cardiac surgery is associated with a more blood product usage, a 2-5 fold increase in the risk of re-exploration and 30-100% increase in the chest drain blood loss. The withdrawal of clopidogrel prior to surgery, between 5 and 7 days, could be associated with a little increase of the risk of myocardial infarction, estimated around 1% while the patient is waiting for surgery [84]. Between patients under aspirin, its withdrawal 2-3 days before surgery could reduce perioperative blood loss, risk for transfusion and reoperation for bleeding. This practice seems safe for patients without acute coronary syndromes, but for urgent cardiac surgery, the risk of perioperative infarction is higher and the balance is favourable to the maintenance of aspirin up to the day of surgery.

So, for patients scheduled for CABG, the recommendation is to stop clopidogrel at least 5 days and, preferably, 10 days prior to surgery to minimize blood loss. In the case of aspirin, the recommendation is to maintain it up to surgery and beyond the time of surgery. But in the case of patients, who are not at high risk for cardiac events, the routine recommendation is to stop aspirin or clopidogrel prior to surgery because of risk of bleeding and morbi-mortality associated in these patients [85].

## Figures and Tables

**Table 1:** Recognized Indications of the Antiplatelet Drugs

**Indications in Cardiology** Acute myocardial infarction Acute coronary syndrome
-Stable angina
-Unstable angina/acute myocardial infarction without Q wave
Percutaneous coronary angioplasty with coronary stent Atrial fibrillation* Patients undergoing CABG surgery Some patients with valvulopathies

**Indications in Neurology** Acute phase of stroke Secondary prevention of strokes in patients without emboligen heart disease

**Other Indications** Patient of valve prosthesis Emboligen carotid stenosis Carotid endarterectomy Patients with Antibodys Antifosfolípidos Peripheral artheriopaty with or without intermittent claudication Primary prevention in patients with cardiovascular risk

* In patients of less than 65 years without another associated risk factor.

**Table 2:** Antiaggregant Effect of Some of the Antiplatelet Drugs

Drug	Complete Reversal Time (days)	Antiaggregant Effect
*Adenosin Diphosphate Receptor Antagonists*		
Ticlopidine	10-14	High
Clopidogrel	7-10	High
*GPIIB/IIIA Receptor Antagonists*		
Eptifibatide	4 hours	High
Tirofiban	4 hours	High
Abciximab	48-72 hours	High
*Inhibitors Of Ciclooxygenase 1 Enzyme (COX-1)*		
ASA	7	High
Piroxicam	7	High
Indometacin	3	High
Ketorolac	2	High
Flurbiprofen	1	High
Ibuprofen	1	Moderate
Naproxen	2	Moderate
Ketoprofen	1	Moderate
Diclofenac	1	Moderate
Salsalate	< 1	Weak
Diflunisal	< 1	Weak
Paracetamol	< 1	Weak
Proparacetamol	< 1	Weak
Metamizol	< 1	Weak
Rofecoxib	0	No
Celecoxib	0	No
*Drugs That Increase The Intraplatelet Levels Of AMPC*		
Trifusal	7	High
Dipyridamole	1	Moderate

**Table 3:** Recommendations on the Use of Antiplatelet Agents

Clinical Setting	Recommendation	Grade

**Ischaemic heart disease**		
Chronic stable angina	Aspirin or clopidogrel (as alternative)	1A
Acute coronary syndrome without ST-segment elevation with PCI	Aspirin or clopidogrel + aspirin (more effective)	1A
	Aspirin or clopidogrel + aspirin (more effective)	
Without PCI	i.v. GPIIb/IIIa inhibitors	1A
	Aspirin	
Acute myocardial infarction with ST elevation	i.v. GPIIb/IIIa inhibitors	2A
		1A
Acute myocardial infarction with ST elevation and with primary PCI		1A

Prior myocardial infarction	Aspirin or clopidogrel (as alternative)	1A

Elective PCI	Aspirin	1A
Elective PCI + stent application	Clopidogrel or ticlopidine	1A
	i.v.GPIIb/IIIa inhibitors	2A

PCI: Percutaneous coronary intervention. Grades of recommendation as defined by Guyatt *et al*. [16].

**Table 4:** Aspirin Withdrawal Preceding Acute Cardiovascular Syndrome

Studies (Author, Year)	Number of Patients Admitted for ACS	% Patients with Recent Withdrawal of APD	% Withdrawal for Surgery	Time between Withdrawal Aspirin and ACS
Collet, 2004	1358	73(5.4%)	74.38%	11.9±0.8
Collet, 2000	475	11(2.3%)	81%	10
Ferrari, 2005	1236	51(4.1%)	13.72%	10±1.9

ACS: acute cardiovascular syndrome

## References

[1] Silber S, Albertsson P, Aviles FF (2005). Guidelines for percutaneous coronary interventions. The Task Force for Percutaneous Coronary Interventions of the European Society of Cardiology. Eur Heart J.

[2] Popma JJ, Berger P, Ohman EM, Harrington RA, Grines C, Weitz JL (2004). Antithrombotic therapy durin percuraneous coronary intervention: The Seventh ACCP Conference on Antithrombotic and Thrombolytic Therapy. Chest.

[3] Bojar RM (2005). Manual of Perioperative Care in Adult Cardiac Surgery.

[4] Collet JP, Montalescot G, Balnchet B (2004). Impact of prior use or recent withdrawal of oral antiplatet agents on acute coronary syndromes. Circulation.

[5] Ia Kovou I, Schmidt T, Bonizzoni E (2005). Incidence, predictors and aoutcomme of thrombosis after successful implantation of drug-eluting stents. JAMA.

[6] Ferrari E, Benhamou M, Cerboni P, Marcel B (2005). Coronary syndromes following aspirin withdrawal: an especial risk for late stent thrombosis. J Am Coll Cardiol.

[7] Samama CM, Bastien O, Forestier F (2002). Antiplatelet agents in the perioperative period: expert recommendations of the French Society of Anesthesiology and Intensive Care (SFAR) 2001 – Summary statement. Can J Anesth.

[8] Lecompte T, Hardy JF (2006). Antiplatelet agents and perioperative bleeding. Can J Anesth.

[9] Patrono C, Coller B, Fitzgerald GA, Hirsh J, Roth G (2004). Platelet active drugs: the relationships among dose, effectiveness and side effects. Chest.

[10] Hirsh J, Warkentin TE, Shaughnessy SG (2001). Heparin and low-molecular-weight heparin. Mechanisms of action, pharmacokinetics, dosing, monitoring, efficacy, and safety. Chest.

[11] L'Allier PL, Ducrocq G, Pranno N (2008). Clopidogrel 600-mg double loading dose achieves stronger platelet inhibition than conventional regimens. Results from PREPAIR randomized study. J Am Coll Cardiol.

[12] Servin F (2007). Low-dose aspirin and clopidogrel: how to act in patients scheduled for day surgery. Curr Opin Anaesthesiol.

[13] Albaladejo P, Marret E, Piriou V, Samama CM (2006). Perioperative management of antiplatelet agents in patients with coornary stents: recommendations of a French Task Force. Br J Anaesth.

[14] Dalal AR, D'Souza S, Shulman RS (2006). Brief review: Coronary drug-eluting stents and anesthesia. Can J Anesth.

[15] (2004). The Task Force on the use of antiplatelet agents in patients with atherosclerotic cardiovascular disease of the European Society of Cardiology. Espert consensus document on the use of antiplatelet agents. Eur Heart J.

[16] Guyatt G, Schunëmann H, Cook D (2001). Grade of recommendation for antithrombotic agents. Chest.

[17] Antithrombotic Trialists' Collaboration (2002). Collaborative meta-analysis of randomized trials of antiplatelet therapy for prevention ofdeath, myocardial infarction, and stroke in high risk patients. BMJ.

[18] Sun JCJ, Whitlock RW, Cheng J (2008). The effect of pre-operative aspirin on bleeding, transfusion, myocardial infarction, and mortality in coronary artery bypass surgery: a systematic review of randomized and observatina studies. Eur Heart J.

[19] Jimenez AH, Stubbs ME, Tofler GH, Winther K, Williams GH, Muller JE (1992). Rapidity and duration of platelet suppression by enteric-coated aspirin in healthy young men. Am J Cardiol.

[20] McDonald JW, Ali M (1983). Recovery of cyclooxigenase activity after aspirin in populations of platelets separated on stractan density gradients. Prostaglandins Leukot Med.

[21] Beving H, Zhao C, Albage A, Ivert T (1996). Abnormally high platelet activity after discontinuation of acetylsalicylic acid treatment. Blood Coagul Fybrinolysis.

[22] Ferrari E, Benhamou M, Cerboni P, Marcel B (2005). Coronary syndromes following aspirin withdrawal, a special risk for late stent thrombosis. J Am Coll Cardiol.

[23] Collet JP, Himbert F, Steg PG (2000). Myocardial infarction after aspirin cessation in stable coronary artery disease patients. Int J Cardiol.

[24] Burguer W, Chemnitius JM, Kneissl GD, Rücker G (2005). Low-dose aspirin for secondary cardiovascular prevention –cardiovascular risks after its perioperative withdrawal versus bleeding risks with its continuation- review a meta-analysis. J Intern Med.

[25] Fox KA, Mehta SR, Peters R (2004). Benefits and risks of the combination of clopidogrel and aspirin in patients undergoing surgical revascularization for non ST-elevtion acute coronary syndrome: th Clopidogrel in Unstable angina to prevent Recurrent ischemic Events (CURE) Trial. Circulation.

[26] Ho PM, Peterson ED, Wang L (2008). Incidence of death and acute myocardial infarction associated with stopping clopidogrel after acute coronary syndrome. JAMA.

[27] Thurston AV, Briant SL (1993). Aspirin and post-prostatectomy haemorrhage. Br J Urol.

[28] Palmer JD, Sparrow OC, Ianotti F (1994). Postoperative hematoma: a 5-year survey and identification of avoidable risk factors. Neurosurgery.

[29] Weightman WM, Gibbs NM, Weidmann CR (2002). The effect of preoperative aspirin-free interval on red blood cell tansfusion requirements in cardiac surgical patients. J Cardiothorac Vasc Anesth.

[30] Lindblad B, Persson NH, Takolander R, Bergqvist D (1993). does low dose acetylsalicylic acid prevent stroke after carotid surgery? A double-blind, placebo-controlled randomized trial. Stroke.

[31] Kallis P, Tooza JA, Talbot S, Cowans D, Bevan DH, Treasure T (1994). Preoperative aspirin decreases platelet aggregation and increases post-operative blood loss –a prospective, randomized, placebo controlled, double-blind clinical trial in 100 patients with chronic stable angina. Eur J Cardiothorac Thorac Surg.

[32] Morawski W, Sanak M, Cisowski M (2005). Prediction of the excessive perioperative bleeding in patients undergoing coronary artery bypass grafting: role of aspirin and platelet glycoprotein IIIa polymorphism. J Thorac Cardiovasc Surg.

[33] Sethi GK, Copeland JG, Goldman S, Moritz T, Zadina K, Henderson WG (1990). Implications of preoperative administration of aspirin in patients undergoing coronary artery bypass grafting. Department of veterans Affairs Cooperative Study on Antiplatelet Therapy. J Am Coll Cardiol.

[34] Rawitscher RE, Jones JW, McCoy TA, Lindsley DA (1991). A prospective study of aspirin’s effect on red blood cell loss in cardiac surgery. J Cardiovasc Surg.

[35] Reich DL, Patel GC, Vela-Cantos F, Bodian C, Lansman S (1994). Aspirin does not increase homologous blood requirements in elective coronary bypass surgery. Anesth Analg.

[36] Vuylsteke A, Oduro A, Cardan E, Latimer RD (1997). Effects of aspirin in coronary artery bypass grafting. J Cardiothorac Vasc Anaesth.

[37] Sun JCJ, Whitlock R, Cheng J (2008). The effect of pre-operative aspirin on bleeding, transfusion, myocardial infarction, and mortality in coronary artery bypass surgery: a systematic review of randomized and observational studies. Eur Heart J.

[38] Alghamdi AA, Moussa F, Fremes SE (2007). Does the use of preoperative aspirin increase the risk of bleeding in patients undergoing coronary artery bypass grafting surgery? Systematic review and meta-analysis. J Card Surg.

[39] Srinivasan AK, Grayson AD, Pullan DM, Fabri BM, Dihmi WC (2003). Effect of preoperative aspirin use in off-pump coronary artery bypass operations. Ann Thorac Surg.

[40] Shim JK, Choi YS, Oh YJ, Bang SO, Yoo KJ, Kwak YL (2007). Effects of preoperative aspirin and clopidogrel therapy n perioperative blood loss and blood transfusion requirements in patients undergoing off-pump coronary artery bypass graft surgery. J Thorac Cardiovasc Surg.

[41] Dacey LJ, Munoz JJ, Johnson ER (2000). Effect of preoperative aspirin use on mortality in coronary artery bypasses grafting patients. Ann Thorac Surg.

[42] Bybee KA, Powell BD, Valeti U (2005). Preoperative aspirin therapy is associated with improved postoperative outcomes in patients undergoing coronary artery bypass grafting. Circulation.

[43] Tuman KJ, McCarthy RJ, O'Connor CJ, McCarthy WE, Ivankovich AD (1996). Aspirin does not increase allogenic blood transfusion in reoperative coronary artery surgery. Anesth Analg.

[44] Karthik S, Grayson AD, McCarron EE, Pullan DM, Desmond MJ (2004). Reexploration for bleeding after coronary artery bypass surgery: risk factors, outcomes, and the effect of time delay. Ann Thorac Surg.

[45] Yende S, Wunderink RG (2001). Effect of clopidogrel on bleeding after coronary artery bypass surgery. Crit Care Med.

[46] Leong JY, Baker RA, Shah PJ, Cherian VK, Knight JL (2005). Clopidogrel and bleeding after coronary artery bypass graft surgery. Ann Thorac Surg.

[47] Kapetantakis EI, Medlam DA, Petro KR (2006). Effect of clopidogrel premedication I off-pump cardiac surgery: are we forfeiting the benefits or reduced hemorrhagic squeal?. Circulation.

[48] Von Heyman, C Redlich, U Moritz M (2005). Aspirin and clopidogrel taken until 2 days prior to coronary artery bypass graft surgery is associated with increased postoperative drainage loss. Thorac Cardiovasc Surg.

[49] Kang W, Theman TE, Reed JF, Stoltzfus J, Weger N (2007). The effect of preoperative clopidogrel on bleeding after coronary artery bypass surgery. J Surg Educ.

[50] Karabulut H, Toraman F, Evrenkaya S, Goksel O, Tarcan S, Alhan C (2004). Clopidogrel does not increase bleeding and allogenic blood transfusion in coronary artery surgery. Eur J Cardiothorac Surg.

[51] Reichert MG, Robinson AH, Travis JA, Hammon JW, Kon ND, Kincaid EH (2008). Effects of a waiting period after clopidogrel treatment before performing coronary artery bypass grafting. Pharmacotherapy.

[52] Bavry AA, Lincoff AM (2006). Is clopidogrel cardiovascular Medicine’s double-edged sword? (Editorial). Circulation.

[53] Pothula A, Sanchala VT, Nagappala B, Inchiosa MA (2004). The effect on preoperative antiplatelet/anticoagulant prophylaxis on postoperative blood loss in cardiac surgery. Anesth Analg.

[54] Kleiman NS (1999). Pharmacokinetics and pharmacodynamics of glycoprotein IIb-IIIa inhibitors. Am Heart J.

[55] Tcheng JE (1999). Differences among the parenteral platelet glycoprotein IIb/IIIa inhibitors and implications for treatment. Am J Cardiol.

[56] Marso SP, Bhatt DL, Roe MT (2000). Enhanced efficacy of eptifibatide administration in patients with acute coronary syndrome requiring in-hospital coronary artery bypass grafting. Circulation.

[57] Dyke CM, Bhatia D, Lorentz TJ (2000). Immediate coronary artery bypass surgery after platelet inhibition with eptifibatatide: results from PURSUIT. Platelet glycoprotein IIb/IIIa in unstable angina: receptor suppression using integrelin therapy. Ann Thorac Surg.

[58] Bizarri F, Scolleta D, Tucci E (2001). Perioperative use of tirofiban hydrocloride (Aggrastat) does not increase surgical bleeding after emergency or urgent coronary artery bypass grafting. J Thorac Cardiovasc Surg.

[59] Shanmugan G (2005). Tirofiban and emergency coronary surgery. Eur J Cardiothorac Surg.

[60] Cheng DK, Jackevivius CA, Seidelin P, Feindel C, Rouleau JL (2004). Safety of glycoprotein IIb/IIIa inhibitors in urgent or emergency coronary artery bypass graft surgery. Can J Cardiol.

[61] Silvestry SC, Smith PK (2000). Current status of cardiac surgery in the abciximab-treated patient. Ann Thorac Surg.

[62] Le Narz LA (2000). Coronary artery bypass in abciximab-treated patients. Ann Thorac Surg.

[63] De Carlo M, Maselli D, Cortese B (2008). Emergency coronary artery bypass grafting in patients with acute myocardial infarction treated with glycoprotein IIb/IIIa receptor inhibitors. Int J Cardiol.

[64] Lincoff AM, LeNArz LA, Despotis GJ (2000). Abciximab and bleeding during coronary surgery: results from the EPILOG and EPISTENT trials. Improve long-term outcome with abciximab GP IIb/IIIa blockade. Evaluation of platelet IIb/IIIa inhibition in STENTing. Ann Thorac Surg.

[65] Sedrakyan A, Treasure T, Elefteriades JA (2004). Effect of aprotinin on clinical outcomes in coronary artery bypass graft surgery: a systematic review and meta-analysis of randomized clinical trials. J Thorac Cardiovasc Surg.

[66] Mangano DT, Tudor IC, Dietzel C (2006). The risk assocaited with aprotinin in cardiac surgery. N Engl J Med.

[67] Schneeweiss S, Seeger JD, Landon J, Walker AM (2008). Aprotinin during coronary-artery bypass grafting and risk of death. N Engl J Med.

[68] Shaw AD, Stafford-Smith M, White WD (2008). The effect of aprotinin on outcome after coronary artery bypass grafting. N Engl J Med.

[69] Have1 M, Grabenwoger F, Schneider J (1994). Aprotinin does not decrease early graft patency after coronary artery bypass grafting despite reducing postoperative bleeding and use of donated blood. J Thorac Cardiovasc Surg.

[70] Kalangos A, Tayyareci G, Pretre R, Di Dio P, Sezerman O (1994). Influence of aprotinin on early graft thrombosis in patients undergoing myocardial revascularization. Eur J Cardiothorac Surg.

[71] Lemmer JH Jr, Stanford W, Bonney SL (1994). Aprotinin for coronary bypass operations: efficacy, safety, and influence on early saphenous vein graft patency-a multicenter, randomized, double-blind, placebo-controlled study. J Thorac Cardiovas Surg.

[72] Pleym H, Stenseth R, Wahba A (2004). Prophylactic treatment with desmopressin does not reduce postoperative bleeding after coronary surgery in patients treated with aspirin before surgery. Anesth Analg.

[73] Casati V, Della Valle P, Benussi S (2004). Effects of tranexamic acid on postoperative bleeding and related hematochemical variables in coronary surgery: comparison between on-pump and off-pump techniques. J Thorac Cardiovasc Surg.

[74] Andreasen JJ, Nielsen C (2004). Prophylactic tranexamic acid in elective, primary coronary artery bypass surgery using cardiopulmonary bypass. Eur J Cardiothorac Surg.

[75] Santos ATL, Kalil RAK, Bauemann C, Pereira JB, Nesralla IA (2006). A randomized, double-blind, and placebo-controlled study with tranexamic acid of bleeding and fibrinolytic activity after primary coronary artery bypass grafting. Braz J Med Biol Res.

[76] Pleym H, Stenseth R, Wahba A, Bjella L, Karevold A, Dale O (2003). Single-dose tranexamic acid reduces postoperative bleeding after coronary surgery in patients treated with aspirin until surgery. Anesth Analg.

[77] Sié P, Steib A (2006). Central laboratory and point of care assessment of perioperative hemostasis. Can J Anesth.

[78] Howard-Alpe GM, de Bono J, Hudsmith L, Orr WP, Foex P, Sear JW (2007). Coronary artery stents and con-cardiac surgery. Br J Anaesth.

[79] Craft RM, Chavez JJ, Snider CC, Muenchen RA, Carroll RC (2005). Comparison of modified thrombelastograph and plateletworks whole blood assays to optical platelet aggregation for monitoring reversal of clopidogrel inhibition in elective surgery patients. J Lab Clin Med.

[80] Lennon MJ, Gibbs NM, Weigthman WM, McGuire D, Michalopoulos N (2004). A comparison of Plateletworks^TM^ and platelet aggregometry for the assessment of aspirin-related platelet dysfunction in cardiac surgical patients. J Cardiothorac Vasc Anesth.

[81] Essell JH, Martin TJ, Salinas J (1993). Comparison of thromboelastography to bleeding time and standard coagulation tests in patients after cardiopulmonary bypass. J Cardiothorac Vasc Anesth.

[82] Hertfelder HJ, Bös M, Weber D, Winkler K, Hanfland P, Preusse CJ (2005). Perioperative monitoring of primary and secondary haemostasis in coronary artery bypass grafting. Semin Thromb Hemost.

[83] Ferraris VA, Ferraris SP, Saha SP (2007). Perioperative blood transfusion and blood conservation in cardiac surgery: The society of thoracic surgeons and society of cardiovascular anesthesiologists clinical practice guideline. Ann Thorac Surg.

[84] Chassot PG, Delabays A, Spanh DR (2007). Perioperative antiplatelet therapy: the case for continuing therapy in patients at risk of myocardial infarction. Br J Anaesth.

[85] Dunning J, Versteegh M, Fabbri A (2008). Guideline on antiplatelet and anticoagulation management in cardiac surgery. Eur J Cardiothorac Surg.

[86] Douketis JD, Berger PB, Dunn AS (2008). The perioperative management of antithrombotic therapy. Chest.

